# Quantitative Analysis of UV-B Radiation Interception in 3D Plant Structures and Intraindividual Distribution of Phenolic Contents

**DOI:** 10.3390/ijms22052701

**Published:** 2021-03-07

**Authors:** Hyo In Yoon, Hyun Young Kim, Jaewoo Kim, Myung-Min Oh, Jung Eek Son

**Affiliations:** 1 Department of Agriculture, Forestry and Bioresources (Horticultural Science and Biotechnology), Seoul National University, Seoul 08826, Korea; yoonhi@snu.ac.kr (H.I.Y.); zosel224@snu.ac.kr (H.Y.K.); plmokn78@snu.ac.kr (J.K.); 2 Division of Animal, Horticultural and Food Sciences, Chungbuk National University, Cheongju 28644, Korea; moh@chungbuk.ac.kr; 3 Brain Korea 21 Center for Bio-Health Industry, Chungbuk National University, Cheongju 28644, Korea; 4 Research Institute of Agriculture and Life Sciences, Seoul National University, Seoul 08826, Korea

**Keywords:** antioxidant activity, *Brassica oleracea*, chlorophyll fluorescence, DPPH, flavonoids, kale, light interception, PSII photochemistry

## Abstract

Ultraviolet-B (UV-B) acts as a regulatory stimulus, inducing the dose-dependent biosynthesis of phenolic compounds such as flavonoids at the leaf level. However, the heterogeneity of biosynthesis activation generated within a whole plant is not fully understood until now and cannot be interpreted without quantification of UV-B radiation interception. In this study, we analyzed the spatial UV-B radiation interception of kales (*Brassica oleracea* L. var. *Acephala*) grown under supplemental UV-B LED using ray-tracing simulation with 3-dimension-scanned models and leaf optical properties. The UV-B-induced phenolic compounds and flavonoids accumulated more, with higher UV-B interception and younger leaves. To distinguish the effects of UV-B energy and leaf developmental age, the contents were regressed separately and simultaneously. The effect of intercepted UV-B on flavonoid content was 4.9-fold that of leaf age, but the effects on phenolic compound biosynthesis were similar. This study confirmed the feasibility and relevance of UV-B radiation interception analysis and paves the way to explore the physical and physiological base determining the intraindividual distribution of phenolic compound in controlled environments.

## 1. Introduction

Ultraviolet (UV) radiation, especially UV-B (280–315 nm), has long been considered a potential stressor for plants, causing damage to DNA, proteins and membranes and reactive oxygen species (ROS) generation due to the higher energy of UV-B quanta [1]. High doses of UV-B, such as a higher fluence rate, longer duration and shorter wavelength, are likely to cause metabolic disorders, such as photosystem II (PSII) photodamage, and ultimately lead to death [2,3,4,5]. In contrast, low doses or ambient levels of UV-B, in which antioxidant capacity is sufficient to deal with the ROS level inflicted by UV-B, initiate numerous acclimation strategies, including changes in plant morphological, physiological, and biochemical traits [6,7,8,9,10]. One of the strategies is the accumulation of secondary metabolite such as phenolic and flavonoid compounds in the epidermis as a direct sun-screening strategy and their innate antioxidant potential to protect sensitive tissues from damage caused by UV-B exposure [11,12,13,14,15]. UV-induced phenolic compounds increase linearly with UV-B dose and absorbance at the leaf level [16,17,18]. The content of secondary metabolites at the whole-plant level is determined depending on the variation in leaf developmental age [19,20]. A typical three-dimensional (3D) plant structure not only consists of leaves at different ages and positions but also causes different UV-B intensities on the leaf surface, leading to the complexity of UV-B-induced responses within the whole plant.

UV exposure patterns in plants rely on both the incident irradiance and absorption, reflection and transmittance of the radiation at the leaf surface or within the leaf, causing UV exposure to diverge into cells, tissues and individual leaves [8,21]. The penetration of UV radiation within a leaf depends on whether sun or shade leaves are present [22]. In other words, UV exposure patterns can be affected by plant architecture and leaf optical properties. However, the actual distribution of light harvest on the plant surface is difficult to measure; thus, the patterns are still poorly understood at the whole-plant level. Recently, light interception analysis based on a 3D plant model has been conducted by ray tracing simulation [23,24,25]. Beyond simplified or virtual plant models, 3D-scanned plant models are sophisticated enough to be used for extracting morphological traits and plant architecture such as leaf width and plant height [26,27] and for estimating the physiological response of plants such as leaf or canopy photosynthesis [28,29]. In particular, 3D-scanned plant models and ray tracing simulations can be applied to analyze the light environment and light interception of leafy vegetables grown under LEDs in controlled environments [30,31]. Thus, the techniques allow quantification of the UV-B light absorbed by each leaf at the whole-plant level.

Even when exposed to the same stress level, the stress susceptibility on each leaf is determined by leaf developmental age. Many studies emphasize the importance of the plant’s developmental stage for its antioxidant capacity and UV stress response [32,33]. For phenolic accumulation within individual plants, the effects of leaf aging and positioning are non-separable [34,35]. Thus, a few studies on the effect of leaf developmental age on the UV response have been conducted in selected plants with a 2D structure, such as horizontal shoots of grapevines and rosettes of *Arabidopsis* [19,20]. Uneven UV-B irradiation and leaf developmental age could result in spatial heterogeneity of plant stress levels and responses within a whole plant. The photochemical activity of PSII, which is sensitive to UV-B exposure, has been measured by chlorophyll fluorescence and is considered to quantify the stress level on plants by various stressors, such as low temperature, drought, UV-A, and UV-B radiation [4,18,36,37,38,39]. Our previous study found that even within an individual plant, leaves more exposed to UV-B showed lower *F_v_*/*F_m_* values and higher contents of total flavonoid compounds [40].

One of the hypotheses in this study is that the plant architecture and optical properties determine the spatial UV-B exposure pattern to the leaf surface within the whole plant. The other is that the leaf developmental age and UV-B level at each leaf codetermine the plant’s response to UV-B. The aim of this study was to reveal the interaction effect of UV-B radiation interception and leaf developmental age on the contents of flavonoids and phenolic compounds within a 3D plant structure in kale. With this aim, radiation interception, PSII photochemical activity, antioxidant capacity, and total phenolic and flavonoid contents were evaluated at different leaf ages within a whole plant.

## 2. Results

### 2.1. Distributions of PAR and UV-B Radiation Interceptions on Kale

**Figure 1:**
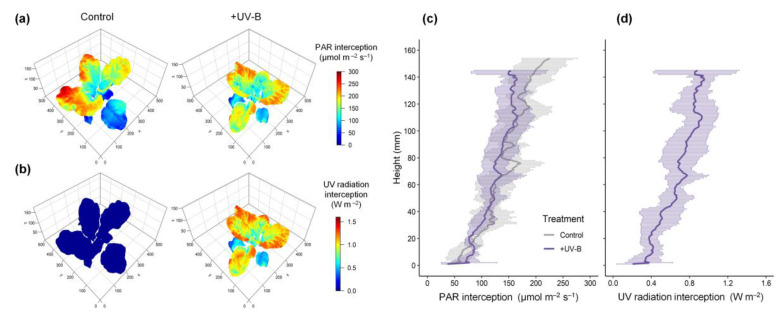
Representative simulated PAR and UV radiation interceptions (**a**,**b**) and vertical distributions (**c**,**d**) on the kales in the control and UV-B treatments. PAR, photosynthetically active radiation in the range of 400–700 nm (**a**,**c**); UV, biological effective UV radiation in the range of 280–400 nm (**b**,**d**). The three axes (x, y, z) represent the actual size in mm. The centerline and area are represented as the mean ± SD (*n* = 3) at each height of the plant model.

The spatial distributions of PAR and UV radiation interception on the 3D kale models were visually depicted along with the actual plant structure, including leaf height, angle, and surface curvature (Figure 1a,b). The PAR and UV radiation interceptions increased with height within the plants regardless of the treatment (Figure 1c,d). On average, the PAR interception on the plants was similar between the control and UV-B treatments (135.85 ± 6.17 and 129.23 ± 4.31 μmol m^−2^ s^−1^, respectively). In the UV-B treatment, the average UV radiation interception was 0.70 ± 0.14 W m^−2^, which is equivalent to 30.32 ± 5.83 kJ m^−2^ d^−1^. The vertical distributions of PAR and UV radiation interceptions showed high linearity (R^2^ = 0.9832, *p* < 0.001, data not shown).

**Figure 2:**
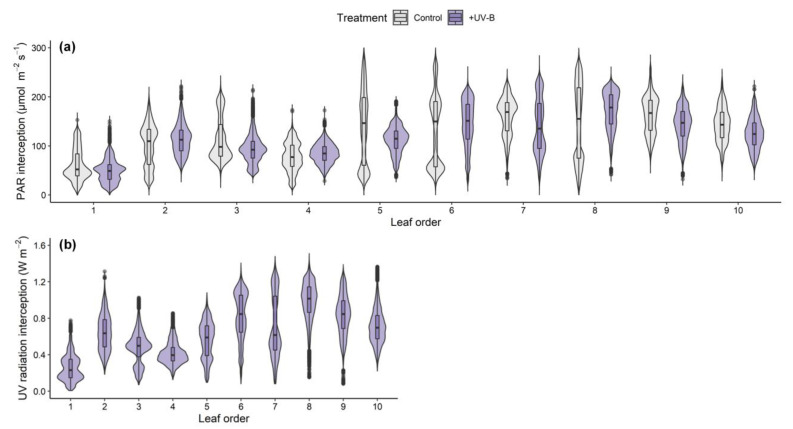
Distributions of PAR (**a**) and UV radiation (**b**) interceptions on the kales at different leaf orders in the control and UV-B treatments. PAR, photosynthetically active radiation in the range of 400–700 nm; UV, biological effective UV radiation in the range of 280–400 nm. The leaf order indicates the order from the oldest leaf at the bottom to the youngest leaf at the top in a whole plant. Based on kernel density estimation, the shape of the violin represents the frequencies of values. The boxes, horizontal lines, whiskers, and points indicate interquartile ranges, medians, 95% confidence intervals, and outliers, respectively.

The distributions of radiation interception at different leaf developmental ages were described with violin plots based on kernel density estimation and box plots (Figure 2). Overall, the median radiation interception was lower in the bottom parts around the 1st–4th leaves and similar between the middle and upper parts. In particular, the distribution in the middle parts could not be characterized. For example, although the median values of the 5th–8th leaves in the control were similar, the bimodal density and longer box indicate that the intercepted light intensity was extremely dispersed at the same leaf age.

### 2.2. Growth Characteristics of Kales According to UV-B and Leaf Order

Both UV-B treatment and leaf developmental age significantly affected the fresh weight and leaf area of individual kale leaves, except for dry weight, which was not affected by UV-B treatment (Table 1). However, when compared at the same leaf developmental age, the three growth parameters at all ages were not significantly different between the control and UV-B treatments. Total growth parameters were also decreased in the UV-B treatment but did not show any significant difference compared to the control (Table 2). Similarly, the total PAR interception per plant did not differ significantly between treatments.

### 2.3. Leaf Photochemistry and Nonphotochemical Quenching

The leaf photochemical efficiency was significantly affected by UV-B treatment or leaf developmental age (Table 3). Both the maximum quantum yield of PSII (*F_v_*/*F_m_*) and the PSII operating efficiency (*Φ_PSII_*) decreased with younger leaves and were lower on average under the UV-B treatment compared to the control. However, the *F_v_*/*F_m_* and *Φ_PSII_* did not show significant differences between treatments at the same leaf age, and the interactions between UV-B treatment and leaf age could not be demonstrated. In contrast, nonphotochemical quenching (*NPQ*) was significantly affected by UV-B treatment, leaf developmental age, and their interaction. Across all data, Pearson’s correlation coefficient between *NPQ* and leaf order was 0.54 at *p* < 0.001 (data not shown), indicating that the *NPQ* increased with younger leaves. Additionally, the *NPQ* was higher under the UV-B treatment than under the control (0.49 and 0.64 on average, respectively). Compared with the control, the increase in *NPQ* in the UV-B treatment was greater with younger leaves, especially in the youngest leaves, in which there was a 2.2-fold increase.

### 2.4. Phenolic Compounds and Antioxidant Capacity

**Figure 3:**
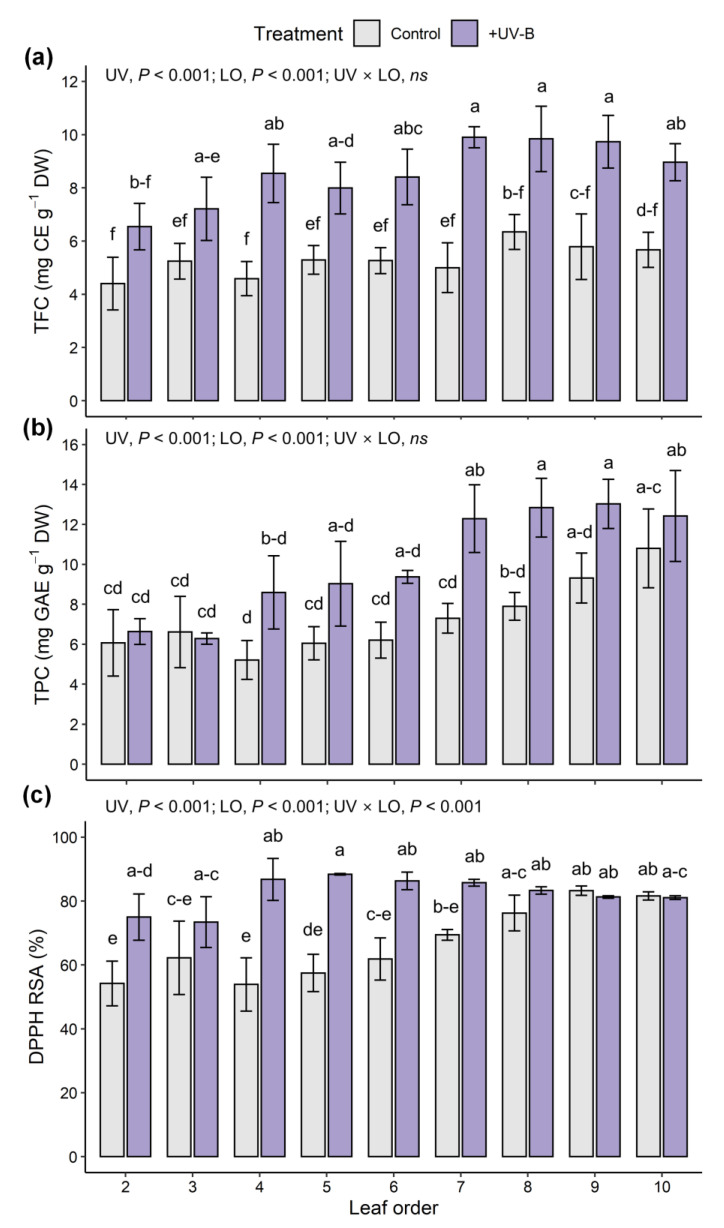
Total flavonoid content (TFC, (**a**)), total phenolic content (TPC, (**b**)), and antioxidant capacity represented as DPPH radical scavenging activity (DPPH RSA, (**c**)) of individual leaves of kales in the control and UV-B treatments. Leaf order indicates the leaf developmental age in order from the oldest leaf at the bottom to the youngest leaf at the top in a whole plant. The *p*-value in two-way ANOVA was represented with UV treatment (UV) and leaf order (LO) and interactions. *ns*, nonsignificance. Different letters indicate significant differences at *p* < 0.05 for each parameter (*n* = 3, mean ± SD) following two-way ANOVA and Tukey’s HSD test.

**Figure 4:**
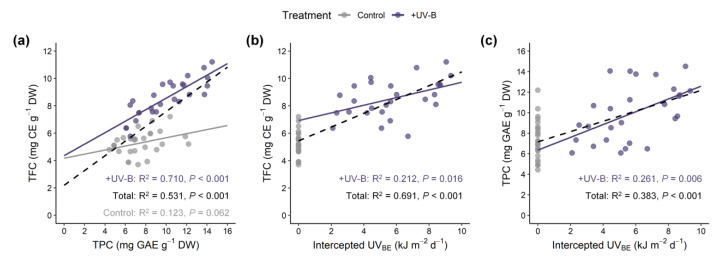
Relationship between total flavonoid content (TFC), total phenolic content (TPC) and UV radiation interception of kale leaves within whole plants grown under the control and UV-B treatment (+UV-B). TPC versus TFC (**a**); UV radiation interception versus TFC (**b**) or TPC (**c**). The lines show linear fits of the +UV-B data set (*n* = 27, purple solid line), the control data set (*n* = 29, gray solid line) and the entire data set (*n* = 56, gray dashed line). The R^2^ and *p*-value for each regression are shown within the panels.

Significant effects of UV-B treatment and leaf developmental age on both TFC and TPC were noted (Figure 3a,b). Compared with the control, TFC in the UV-B treatment increased by 37.6% in UV-exposed leaf 3 and 98.2% in leaf 7. TPC in the UV-B treatment did not increase in UV-exposed leaf 2, but increased by 68.4% in leaf 7 compared with the control. Across all data, TPC was positively correlated with TFC, and their Pearson’s correlation coefficient was 0.73 at *p* < 0.001 (Figure 4a). Similarly, both TFC and TPC were positively correlated with UV radiation interception on individual leaves, and their correlation coefficients were 0.83 and 0.58 at *p* < 0.001, respectively (Figure 4b,c). The AOC measured by the DPPH method was significantly affected by UV-B treatment, leaf developmental age, and their interaction (Figure 3c). The AOC increased with younger leaves and was higher in the UV-B treatment than in the control (66.7% and 82.4% on average, respectively). Compared with the control, the increase in antioxidant capacity was greater with older leaves, especially in UV-exposed leaves, which exhibited a 1.6-fold increase in capacity.

**Figure 5:**
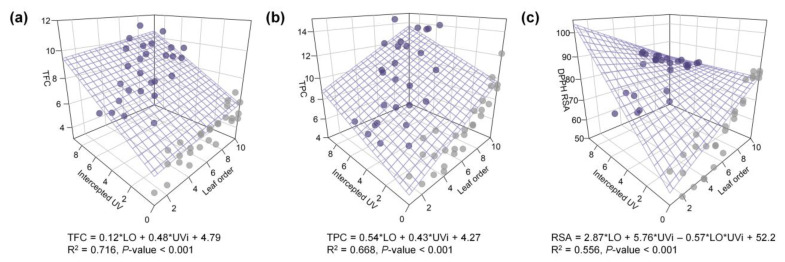
Multiple regression models for total flavonoid content (TFC, mg CE g^−1^ DW, (**a**)), total phenolic content (TPC, mg GAE g^−1^ DW, (**b**)) and antioxidant capacity represented as DPPH radical scavenging activity (RSA, %, (**c**)) with leaf order and biological effective UV radiation interception (kJ m^−2^ d^−1^). The plane or surface showed linear or nonlinear regression model of the entire data set (*n* = 56), and the regression equation, R^2^, and *p*-value are shown below the graph.

Multiple regression models for TFC and TPC and AOC with leaf developmental age and UV radiation interception were developed and showed high explanatory power (Figure 5). The standardized regression coefficients of leaf order (LO) and UVi for TFC were 0.16 and 0.80, respectively, indicating that the effect of UV radiation interception on TFC was 4.9-fold greater than that of leaf age. Those for TPC were 0.54 and 0.53, respectively, indicating similar effects of UV radiation interception and leaf age on TPC. Similar to the results with and without UV-B treatment, the regression model for RSA regressed well with the interaction between UV-B and leaf age (Figure 3c and Figure 5c). All estimated coefficients were significant for the regression models.

## 3. Discussion

The effect of supplemental UV-B radiation on 3D plant structure and its physiological interaction could help to understand how this spatial relationship can affect the contents of antioxidant phenolic compounds in plants. As hypothesized in this study, the plant structure and optical properties of the leaves determined the spatial UV-B absorbed by the leaf surface within the whole plant. It was confirmed that the developmental age and intercepted UV-B level of each leaf co-determined the UV-B-induced accumulation of phenolics, and statistical analyses of each factor were possible through quantification of UV-B radiation interception. The short-term pre-harvest UV-B exposure improved phenolic contents without negative effects on growth and photosynthesis in kale, which is consistent with previous research in basil [41].

### 3.1. Leaf Morphology, Optical Property and UV-B Radiation Interception

**Figure 6:**
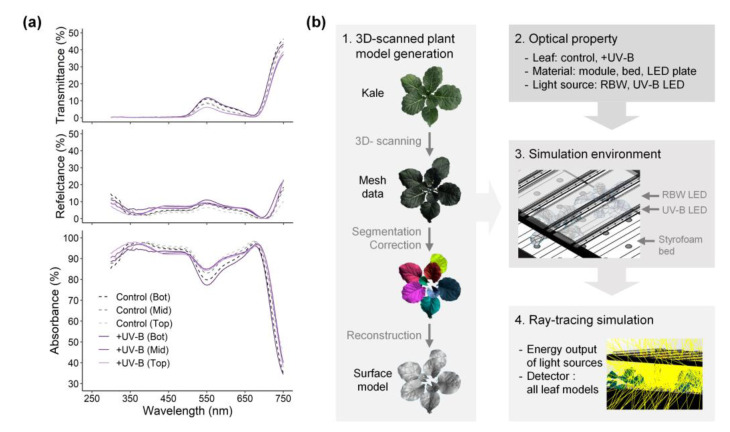
(**a**) Optical properties of kale leaves measured individually at the top (Top), middle (Mid), and bottom (Bot) of the plant in each treatment with three replicates; (**b**) Schematic diagram of radiation interception analysis from 3D scanning to simulation.

UV-B radiation induces changes in whole leaf morphology, such as shorter petioles, shorter stems, and thicker and smaller leaves, which can affect radiation interception in canopy structure [8,42]. In this study, the UV-B treatment also induced smaller leaves than the control (Table 1), which is consistent with previous studies [43,44,45]. However, the low and short-term UV-B doses did not cause noticeable changes in total leaf area or PAR interception (Table 2, Figure 1a,c and Figure 2a). UV-screening pigments accumulate in the epidermis to absorb UV radiation and avoid UV-induced damage [10,12,45,46]. Similarly, the absorbance in the range of 280–350 nm was higher in the upper leaves in the UV-B treatment than in the control (Figure 6a).

The effects of UV-B radiation on leaf morphology and optical properties could be comprehensively considered through a 3D-scanned plant model and simulation parameters in the radiation interception analysis (Figure 6b). In this study, both the PAR and UV radiation interceptions increased with height but varied with leaf order (Figure 1c,d and Figure 2). The phyllotaxis of the kale is a spiral pattern, which is a common pattern, and most often, the divergence angle is close to the golden angle of *c*. 137.5°. The distribution of radiation interception depending on leaf order, shown in Figure 2, was consistent with the patterns of simulated light capture efficiency in digitized A. thaliana leaves placed at a divergence angle of 137.5° [47]. In addition, this arrangement could minimize the overlapping leaf area to maximize light capture, which may cause the linear distribution of radiation interception with height in the kales [47]. The leaf height and arrangement due to divergence angle, i.e., plant structure, caused within-individual heterogeneity of PAR and UV-B exposures regardless of leaf age.

The PAR and UV-B radiation interception at the upper leaf without shade was not greater than the light intensity set at the bottom in this experimental condition (Figure 1), due to the physical light distribution (PLD) of the LEDs, lighting distance from the light sources, and leaf angles. The used LEDs, placed c. 15 cm above the plant, had a wider PLD with a beam angle of 120°, which caused the overlap of light between multiple LED modules. In an empty growth chambers, the light intensity at a distance of 15 cm from the LED modules was lower than that at the longer distance (>30 cm) [30]. With plants in the LED growth chamber, the total light interception of lettuce increased as the lighting distance increased up to 30 cm, i.e., *c*. 20 cm above the plant [31]. That is, when an artificial light source for plant cultivation such as LED is closely irradiated, the plant can receive less light. Even if the PLD and lighting distance are same, the more light is received when the light is incident perpendicular to the leaf surface. In leaves with high curvature, the difference in light interception according to the incident light angle is more noticeable [28]. Therefore, the upper leaves with high curvature and leaf inclination angle may receive much less light than expected.

### 3.2. The Effect of UV-B on Photosynthetic Characteristics

Many early studies on UV-B have reported negative effects on photosynthetic reactions, including chlorophyll fluorescence, CO_2_ fixation and stability of the D1 and D2 proteins of PSII; however, at ambient or low UV-B levels, the effects are less sensitive than expected [6,7,46,47,48,49,50]. Such low UV-B effects are at least partially mediated by the UV-B specific UV RESISTANCE LOCUS 8 (UVR8) photoreceptor and signaling pathway with CONSTITUTIVE PHOTOMORPHOGENESIS1 (COP1) [6,51,52]. In this study, the low and short-term UV-B doses did not cause noticeable damage to the PSII photochemistry of kale but affected nonphotochemical quenching (Table 3). *NPQ*, also called energy-dependent quenching, qE, is the major mechanism of photoprotection, which allows thermal dissipation of excess energy [10]. After UV-B exposure, qE is regulated by UVR8-mediated signaling together with COP1 [53]. In the present result, although there was no significant difference in UV radiation interception between the upper leaves, the significant increase in *NPQ* in the youngest leaves was presumed to be due to age-dependent susceptibility to UV-B exposure.

### 3.3. The Effect of UV-B Radiation on Phenolic and Flavonoid Compounds

UV-B-induced phenolic and flavonoid compounds were analyzed with the UV radiation interception and leaf developmental age of kale leaves at the whole-plant level (Figure 3, Figure 4 and Figure 5). As a response to UV-B, the enhanced biosynthesis of flavonoids and related phenolic compounds has been well documented in various plants, such as basil, blueberry leaves, broccoli, lettuce and wheat [13,39,54,55,56], which are regarded as protective mechanisms against enhanced UV-B exposure. The accumulated phenolics act as UV-screen pigments in epidermal tissues and increase their innate antioxidative potential for scavenging ROS generated under UV-B exposure [12,15,46]. The signal transduction of UVR8–COP1 with the ELONGATED HYPOCOTYL5 (HY5) transcription factor plays a central role in the regulation of genes involved in controlling the biosynthesis of UV-protective flavonoid and phenolic compounds [6,52,57]. In this study, the UV-B_BE_ dose of 4.2 kJ m^−2^ d^−1^ was sufficient to improve TFC and TPC, and the contents increased with the amount of intercepted UV-B in the range of 2–10 kJ m^−2^ d^−1^ (Figure 4b,c). Similarly, a previous study reported that supplemental UV-B (ambient + 3.6 kJ m^−2^ d^−1^ of UV-B_BE_) increased the contents of flavonoids and phenolics and enhanced the activities of phenylpropanoid pathway enzymes in the medicinal plant *Coleus forskohlii* [9]. UV-B-induced phenolic biosynthesis also depends on background light, i.e., PAR intensity [58]. In this study, the TPC was positively correlated with PAR interception (*r* = 0.33 at *p* < 0.05), but the TFC was not (data not shown). In the UV-B treatment, the linear regression slope of TPC versus TFC was higher than that in the control, which may be interpreted as flavonoid biosynthesis being more stimulated among the related phenolic compounds induced by UV-B exposure (Figure 4a).

### 3.4. Leaf Age as a Physiological Factor Determining UV-B Induced Phenolic Content

Leaf developmental age had a significant impact, such as higher TPC and AOC in younger leaves, but did not affect the TFC of kales without UV-B exposure (Figure 3). The relationship between leaf developmental age and phenolic content was not straightforward, as the content may increase, decrease, or not change with leaf age [20]. However, the increases in TFC and TPC were higher with younger leaves in this study. Similarly, the quercetin contents were increased more by UV-B exposure in young leaves compared with the older leaves of two Brassicaceae, *Sinapis alba* and *Nasturtium officinale* [59]. In Gingko, younger leaves showed distinct increases in quercetin and kaempferol contents and were more sensitive to UV-B radiation than old leaves [60]. In grapevines, UV-B-exposed younger leaves showed increases in UV-absorbing pigments, phenolics, and antioxidant capacity compared with older leaves [19]. Particularly in primary leaves of barley, flavonoids are needed for efficient UV-B protection without changes in variable chlorophyll fluorescence [61]. These previous studies showed that UV-B-induced antioxidant responses are dependent on leaf developmental age. In contrast, the antioxidant capacity was increased in older leaves from leaves 2 to 6 (Figure 3c). The increases in phenol and flavonoid contents in young leaves led to an increase in antioxidant activity, but in this experiment, the measured value at the upper younger leaves was saturated, and no increase was observed.

### 3.5. Potential for Estimating UV-B Induced Phenolic Content in 3D Plant Structure

**Figure 7:**
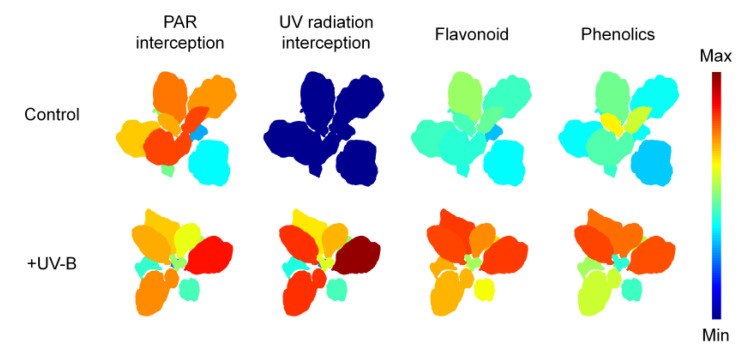
Representative distributions of average radiation interception and phenolic compound contents on individual leaves of kales under the control and UV-B treatments (*n* = 3). PAR, photosynthetically active radiation in the range of 400–700 nm; UV, biological effective UV radiation in the range of 280–400 nm. The color var indicates each range of 0–200 μmol m^−2^ s^−1^ for PAR interception, 0–10 kJ m^−2^ d^−1^ for UV radiation interception, 0–12 mg CE g^−1^ DW for flavonoids and 0–16 mg GAE g^−1^ DW for phenolic compounds.

Multiple regression analyses for TFC and TPC and RSA with leaf age and intercepted UV were conducted to assess their relationships considering the effect of leaf positioning (Figure 5). Although the experimental UV-B dose was the same, the heterogeneous UV-B doses depending on leaf position were reflected as UV radiation interception. UV-B interception and leaf age were each linearly involved in UV-induced phenolic compounds, and their determinant power was dependent on the type of compound. Dose- and structure-dependent responses of phenolic compounds were reported in kales exposed to short-term and moderate UV-B radiation [62]. In long-term adaptation, the sensitivity/susceptibility of plants to the overall UV-B response is also determined by plant growth attributes, such as growth rate, epidermal cell surface, and accumulated UV-B-absorbing compound level [63]. The data presented here suggest that the intraindividual distribution of antioxidant phenolic contents under low and short-term UV-B radiation can be estimated by UV radiation interception with leaf developmental age. UV-B exposure further enhanced the intraindividual heterogeneity of TFCs and TPCs within the 3D plant structure compared with those without UV-B radiation (Figure 7). As a further study, it is necessary to analyze the determinants for the distribution of UV-induced metabolites with various growth stages and canopy levels.

Overall, mild and short-term UV-B radiation did not cause noticeable changes in growth and photochemical activity in PSII but promoted TFC and TPC and enhanced the antioxidant capacities of kale. UV radiation interception was determined by plant architecture and leaf optical properties and was quantified with a 3D plant model and ray-tracing simulation. The spatial distributions of phenolic compounds were more heterogeneous within the whole plant under UV-B exposure. The intraindividual distributions of phenolic compounds could be determined by UV radiation interception and leaf age, and their determinant power was dependent on the type of compounds. These quantitative approaches could contribute to finding optimal UV-B dose levels and estimating the bioactive compound contents without harm to even whole plants from a practical point of view. In addition to the accumulation of secondary metabolites, the various plant responses to UV-B radiation will be better understood in 3D plant structures through light interception analysis.

## 4. Materials and Methods

### 4.1. Plant Material and Growth Condition

**Figure A1:**
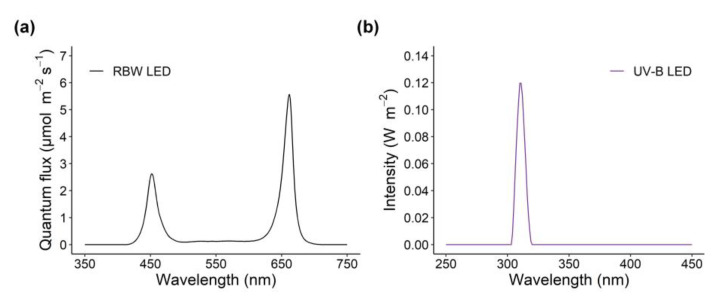
Light spectra of the light source. (**a**) Red, blue and white LEDs (RBW LED) at a photosynthetically active radiation of 200 μmol m^−2^ s^−1^; (**b**) UV-B LED with a spectrum peak at 310 nm at a dose of 1.0 W m^−2^.

Kale seeds (*Brassica oleracea* L. var *Acephala*) were sown on sponge cubes in water culture under fluorescent lamps at a photosynthetic photon flux density (PPFD) of 150 μmol m^−2^ s^−1^ over the waveband 400–700 nm for 16-h light periods. The nutrient solution for *Brassica* was applied with an electrical conductivity (EC) of 0.6 dS m^−1^ after normal leaves appeared. After the fourth normal leaf appeared, seedlings of uniform size were transplanted into plant factory modules with a deep flow technique system and were maintained at an air temperature of 20 °C, a relative humidity of 70%, and a CO_2_ concentration of 500 μmol mol^−1^. The plants were irradiated with red, blue and white light-emitting diodes (RBW LEDs) at a PPFD of 200 μmol m^−2^ s^−1^ for 16-h light periods. The spectrum of the RBW LED was measured using a spectroradiometer (Blue-Wave spectrometer, StellarNet Inc., Tampa, FL, USA) in the range of 380–900 nm (Figure A1). The nutrient solution was supplied with an EC of 1.2 dS m^−1^. The plants were harvested at 26 days after transplanting (DAT).

### 4.2. UV Treatment

Enhanced UV-B radiation was produced by UV-B LED with spectrum peak *c*. 310 nm, which consisted of five bar module arrays containing twelve chips per bar (Ericsong Company Ltd., Bucheon, Korea). The plants were exposed to UV-B LEDs for 12 h per day for two days (+UV-B) with the RBW LEDs from the start of the light period and were harvested after recovery for 4 h. The UV-B dose was 1.0 W m^−2^ (43.2 kJ m^−2^ d^−1^) at the bottom, which is equivalent to a biologically effective UV radiation (UV_BE_) dose of 4.4 kJ m^−2^ d^−1^ (UV-B_BE_ = 4.2, UV-C_BE_ = 0.0 and UV-A_BE_ = 0.2 per day). UV_BE_ was calculated using a plant action spectrum in the UV range [64]. The light intensity and spectrum of the UV-B LED were measured with a UV sensor (MU-200, Apogee Instruments Inc., Logan, UT, USA) and the spectroradiometer in the range of 280–400 nm (Figure A1).

### 4.3. Chlorophyll Fluorescence Imaging

**Figure A2:**
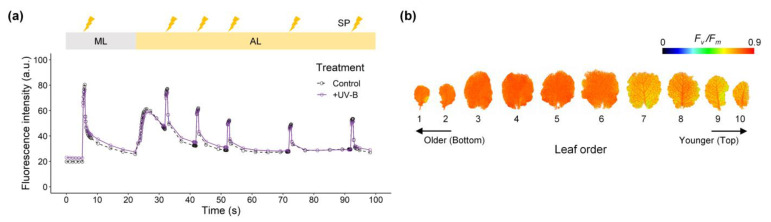
Representative kinetic chlorophyll fluorescence curves (**a**). ML, measuring light (a short flash of very weak intensity, 5 s duration, <0.5 μmol m^−2^ s^−1^); AL, actinic light; and SP, saturating pulses (800 ms duration, 1200 μmol m^−2^ s^−1^). (**b**) The chlorophyll fluorescence parameter images according to the leaf order: *F_v_*/*F_m_*, the maximum quantum yield of PSII (Equation (1)). The leaf order indicates the order from the oldest leaf at the bottom to the youngest leaf at the top in a whole plant.

Chlorophyll fluorescence (ChlF) images of each leaf were obtained by quenching analysis using a closed chlorophyll fluorescence imaging system (FluorCam 800MF, Photon Systems Instruments, Brno, Czech Republic), and its software (FluorCam 7, Photon Systems Instruments) was used to control the image system and to process the images. All leaves were measured separately at harvest after the recovery time with three replicates. The fluorescence was detected by a high-sensitivity CCD camera, generating 1360 × 1024-pixel images with 16-bit resolution. The light source included two red-orange LED panels (617 nm) for measuring light (a short flash of very weak intensity) and actinic light and another pair of cool white LED panels (6500 K) for saturating pulses with an angle of 45°. The detailed ChlF quenching protocol is described in Figure A2. After 20 min of dark adaptation, the minimal fluorescence (*F_o_*) was determined with a measuring light (5 s duration, <0.5 μmol m^−2^ s^−1^), followed by a saturating light pulse (800 ms duration, 1200 μmol m^−2^ s^−1^) so that the maximal fluorescence in the dark-adapted state (*F_m_*) was recorded. After dark relaxation for 17 s, the leaves were exposed to actinic light (70 s duration, 600 μmol m^−2^ s^−1^). The instantaneous fluorescence during light adaptation was measured before any of the saturating pulses and later declined to steady-state fluorescence in light (*F*′). The saturating pulses were applied 5 times, determining the maximum fluorescence during light adaptation and at the light-adapted steady state (*F*′*_m_*).

All images of ChlF parameters for each leaf on the whole plant were individually obtained using imaging system software (Figure A2). Based on the basic ChlF signals, the maximum quantum yield of PSII (*F_v_*/*F_m_*), nonphotochemical quenching (*NPQ*), and PSII operating efficiency (*Φ_PSII_*) can be derived as a set of equations [65,66]:

*F_v_*/*F_m_* = (*F_m_* − *F_o_*)/*F_m_* (1)

*NPQ* = (*F_m_* − *F*′*_m_*)/*F*′*_m_* (2)

*Φ_PSII_* = (*F’_m_* − *F*′)/*F*′*_m_* (3)

A total of 250 ChlF images and kinetics were acquired. Data preprocessing and region-of-interest (ROI) selection were performed using the imaging system software, including segmentation for background exclusion and averaging within the ROI. The ROI was semi-automatically selected based on the same criteria, such as minimum size of selected pixels and selection range of signal level so that the entire leaf becomes one ROI. For statistical analysis, the mean and standard deviation of each parameter were obtained and analyzed.

### 4.4. Radiation Interception on Plant

#### 4.4.1. D-Scanned Plant Model Generation

Plant models were directly obtained by 3D scanning of three plants per treatment using a high-resolution portable 3D scanner (Go! SCAN50TM, Creaform, Lévis, QC, Canada) with a resolution of 2 mm, and its scan software (Vxelement, Creaform) was used to record and export the scan data to mesh data. The 3D mesh data were corrected for holes and noise and split into leaf mesh data. The segmented mesh data were then reconstructed into individual surface models for ray tracing simulation using reverse engineering software (Geomagic Design X, 3D Systems, Rock Hill, SC, USA). The constructed 3D plant models were transferred to 3D CAD software (Solidworks, Dassault Systèmes, Vélizy-Villacoublay, France). The procedure from scan to simulation is described in Figure 6b [28].

#### 4.4.2. Optical Properties and Simulation Environment

The spectral transmittance (Tr) and reflectance (Ref) of leaves were measured using the spectroradiometer at 1-nm resolution with a 50-mm integrating sphere (IC-2, StellarNet Inc., Tampa, FL, USA), and the optical properties of their 3D models were determined. The measurements were carried out on 9 leaves (from three plants) per treatment, according to the leaf positions sampled at the top, middle, and bottom (Figure 6a). In the same way, the optical properties of the materials within the cultivation environment, such as the plant factory module, styrofoam bed, RBW and UV-B LED plate, were measured.

For simulation, the materials of the cultivation environment were implemented as a 3D virtual environment using 3D CAD software with the same size and layout as the actual environment. Spectral power distributions of RBW and UV-B LEDs were set with the measured spectra, and the physical light distribution was set as a Lambertian distribution with a half angle of 60°. All 3D models were placed in the same position and orientation as the actual materials and plants.

#### 4.4.3. Ray Tracing Simulation

The ray tracing simulation was performed using ray tracing software (Optisworks, Optis Inc., La Farlède, France) with a total of 500 mega-rays. To match the light intensity in the virtual environment with the actual light intensity, a cylinder-shaped detector was modeled and placed based on a quantum sensor. The energy outputs of light sources were set to 7.1017 W for RBW LED plates and 0.0176 W for UV-B LED chips, representing a PPFD of 200 μmol m^−2^ s^−1^ and a UV intensity of 1.0 W m^−2^, respectively. All leaf surface models were set up as separate detectors, and all simulations of the treatments were performed under the same conditions. The simulation results are represented as photosynthetically active radiation (PAR) interception in the range of 400–700 nm and UV radiation interception in the range of 280–400 nm (UVi).

### 4.5. Phenolic Compounds and Antioxidant Capacity

#### 4.5.1. Sample Extraction

All leaves of three plants per treatment were sampled and lyophilized using a freeze dryer (FD8512, Ilshin Biobase Co., Yangju, Korea) at −80 °C under a vacuum of 0.007 mmHg for 120 h. The 50-mg freeze-dried samples were ground and extracted with 1 mL of 70% (*v*/*v*) methanol and 2.8-mm ceramic beads using a bead mill homogenizer (Beadruptor 4, Omni International, Kennesaw, GA, USA).

#### 4.5.2. Total Flavonoid Content

The total flavonoid content (TFC) was measured by the aluminum chloride colorimetric method [67]. The extract solution was incubated for 12 h in the dark at 4 °C and then centrifuged at 1.0 × 10^4^ g for 10 min. The supernatant (150 μL) was collected in a 2-mL microtube, and 135 μL of distilled water and 45 μL of 5% NaNO_2_ were added. After 5 min, 90 μL of 10% AlCl_3_ was added. After an additional 5 min, 300 μL of 1 M NaOH and 165 μL of distilled water were added, and all reactants were thoroughly mixed. After incubating for 6 min, the absorbance of the samples was read at 510 nm using a spectrophotometer (Photolab 6100vis, WTW, Weilheim, Germany), and the standard unit was expressed as milligrams of catechin acid (Supelco, Bellefonte, PA, USA) equivalent per gram of dry weight (mg CE g^−1^ DW).

#### 4.5.3. Total Phenolic Content

Total phenolic content (TPC) was measured by the Folin-Ciocalteu colorimetric method [68]. The extract solution was incubated for 48 h in the dark at room temperature and then centrifuged at 1.0 × 10^4^ g for 10 min. The supernatant (50 μL) was collected in a 2-mL microtube, and 750 μL of 10% Folin–Cioalteu solution (Junsei Chemical Co. Ltd., Tokyo, Japan) and 135 μL of distilled water were added. After vortex mixing, 600 μL of 700 mM Na_2_CO_3_ was added, followed by incubation for 2 h at room temperature. The absorbance of the samples was read at 765 nm using the spectrophotometer, and the standard unit was expressed as milligrams of gallic acid (Sigma-Aldrich, St. Louis, MO, USA) equivalent per gram of dry weight (mg GAE g^−1^ DW).

#### 4.5.4. Antioxidant Capacity

Antioxidant capacity (AOC) was measured using a 2,2-diphenyl-1-picrylhydrazyl (DPPH) assay [69]. The DPPH was purchased from Alfa Aesar (Ward Hill, MA, USA). The extract solution was incubated for 48 h in the dark at room temperature and then centrifuged at 1.0 × 10^4^ g for 10 min. The supernatant (100 μL) was collected in a 2-mL microtube, and 1.25 mL of 6 × 10^−6^ M DPPH MeOH solution was added. After reaction for 30 min, the absorbance (*A*) values of the samples and blank were read at 517 nm. The percentage of DPPH radical scavenging activity was calculated as follows:

DPPH radical scavenging activity (%) = (*A_blank_* − *A_sample_*)/*A_blank_* × 100 (4)

### 4.6. Plant Growth Characteristics

The fresh weight of each leaf was measured at harvest, and the dry weight was measured after drying in an oven at 70 °C for 72 h. Leaf developmental age was numbered in order from the oldest leaf at the bottom to the youngest leaf at the top, ranging from 1 to 10 in all samples (Figure A2). The leaf area of individual leaves was obtained from the mesh data of the 3D plant model using reverse engineering software.

### 4.7. Statistical Analysis

All visualization and statistical analyses were performed using R software (R 3.6.2, R Foundation, Vienna, Austria). Comparisons of the mean trait values between treatments were performed with two-way ANOVA and Tukey’s HSD test to assess the effects of the UV-B treatment, leaf developmental age, and their interactions. Pearson’s correlation coefficients and multiple regression were conducted.

## Figures and Tables

**Table 1:** Growth parameters of individual kale leaves grown under the control and UV-B exposure conditions for 2 days before harvest.

Leaf Order ^1^	Leaf Fresh Weight (g)		Leaf Dry Weight (g)		Leaf Area (cm^2^)	
	Control	+UV-B	Control	+UV-B	Control	+UV-B
2	2.36 ± 1.54 ^2^	2.66 ± 0.40	0.18 ± 0.11	0.21 ± 0.01	32.25 ± 9.65	45.23 ± 4.16
3	5.15 ± 2.84	4.87 ± 1.39	0.41 ± 0.26	0.38 ± 0.08	84.49 ± 49.35	87.24 ± 28.17
4	8.31 ± 1.55	8.57 ± 0.99	0.70 ± 0.20	0.82 ± 0.19	168.31 ± 34.38	155.03 ± 13.80
5	8.97 ± 1.33	8.44 ± 1.25	0.88 ± 0.14	0.86 ± 0.15	179.44 ± 16.96	186.87 ± 14.14
6	10.17 ± 2.56	8.62 ± 0.69	0.93 ± 0.33	0.89 ± 0.17	200.07 ± 36.42	178.14 ± 13.40
7	10.12 ± 1.09	7.06 ± 1.97	0.96 ± 0.21	0.79 ± 0.29	182.72 ± 19.97	147.06 ± 28.89
8	7.40 ± 1.76	5.80 ± 1.54	0.77 ± 0.21	0.66 ± 0.18	139.49 ± 17.85	115.14 ± 33.49
9	5.38 ± 0.86	4.18 ± 1.05	0.57 ± 0.09	0.50 ± 0.12	91.20 ± 1.82	64.23 ± 19.42
10	3.59 ± 0.53	2.82 ± 0.50	0.43 ± 0.08	0.37 ± 0.06	49.94 ± 6.36	37.74 ± 14.75
UV treatment		* ^3^		*ns*		*
Leaf order		***		***		***
UV × Leaf order		*ns*		*ns*		*ns*

^1^ Leaf order indicates the leaf developmental age in order from the oldest leaf at the bottom to the youngest leaf at the top in a whole plant. ^2^ Data represent mean ± SD (*n* = 3). ^3^ Asterisks indicate significant differences following two-way ANOVA and Tukey’s HSD test. *, *p* < 0.05; ***, *p* < 0.001; *ns*, not significant.

**Table 2:** Total growth and radiation interception of kales grown under the control and UV-B exposure conditions for 2 days before harvest.

Treatment	Total Growth Parameter			Total Radiation Interception Per Plant	
	Fresh Weight (g)	Dry Weight (g)	Leaf Area (cm^2^)	PAR ^2^ (mmol Plant^−1^ d^−1^)	UV_BE_^3^ (MJ Plant^−1^ d^−1)^
Control	79.5 ± 14.0 ^1^	7.39 ± 1.83	1142.5 ± 184.8	914.33 ± 209.50	-
+UV-B	67.8 ± 6.3	6.91 ± 0.94	1006.0 ± 67.6	739.48 ± 102.24	2.91 ± 0.47

^1^ Data represent mean ± SD (*n* = 3). The mean difference between treatments was not significant. ^2^ PAR, photosynthetically active radiation in the range of 400–700 nm. ^3^ UV_BE_, biological effective UV radiation in the range of 280–400 nm.

**Table 3:** Chlorophyll fluorescence parameters of individual leaves of kales under the control and UV-B exposure conditions for 2 days before harvest: *F_v_*/*F_m_*, the maximum quantum yield of PSII; *Φ_PSII_*, the PSII operating efficiency; *NPQ*, nonphotochemical quenching.

Leaf Order ^1^	*F_v_*/*F_m_*		*Φ_PSII_*		*NPQ*	
	Control	+UV-B	Control	+UV-B	Control	+UV-B
2	0.76 ab ^2^	0.77 a	0.57 abc	0.61 ab	0.49 b	0.40 b
3	0.78 a	0.78 a	0.62 a	0.58 abc	0.39 b	0.45 b
4	0.78 a	0.78 a	0.56 abcd	0.52 bcde	0.65 b	0.47 b
5	0.78 a	0.77 a	0.50 cdef	0.47 defg	0.45 b	0.47 b
6	0.78 a	0.76 a	0.46 efg	0.43 fg	0.43 b	0.55 b
7	0.74 abc	0.71 abcd	0.44 efg	0.42 fg	0.45 b	0.71 ab
8	0.74 abc	0.68 bcd	0.44 fg	0.42 fg	0.49 b	0.57 b
9	0.71 abcd	0.67 cd	0.45 efg	0.41 g	0.49 b	0.85 ab
10	0.72 abcd	0.66 d	0.48 efg	0.41 g	0.57 b	1.27 a
UV treatment		**		*		**
Leaf order		***		***		**
UV × Leaf order		*ns*		*ns*		*

^1^ Leaf order indicates the leaf developmental age in order from the oldest leaf at the bottom to the youngest leaf at the top in a whole plant. ^2^ Different letters indicate significant differences at *p* < 0.05 for each parameter (*n* = 3, mean) following two-way ANOVA and Tukey’s HSD test. *, *p* < 0.05; **, *p* < 0.01; ***, *p* < 0.001; *ns*, not significant.

## Data Availability

Data is contained within the article.
